# Supplementary material for: Neutrophil-Lymphocyte Ratio in Patients with Acute Heart Failure Predicts In-Hospital and Long-Term Mortality
Source: J Clin Med. 2020 Feb 18;9(2):557. doi: 10.3390/jcm9020557 (PMC7073552; doi:10.3390/jcm9020557)
Supplement: Supplementary file 1 [file jcm-09-00557-s001.zip › NL_ratio_supple table_JCM_revision_final.docx]

**Supplementary Table 1.** In-hospital Clinical Outcomes Stratified by quartiles of neutrophil to lymphocyte ratio at admission.

|  | **Total population** | **Neutrophil to lymphocyte ratio** | | | | |
| --- | --- | --- | --- | --- | --- | --- |
|  | (n = 5,580) | **Quartile 1**  (n = 1,395) | **Quartile 2**  (n = 1,395) | **Quartile 3**  (n = 1,395) | **Quartile 4**  (n=1,395) | **P-value** |
| Total mortality, N (%)  (including urgent heart TPL) | 331 (5.9%) | 58 (4.2%) | 52 (3.7%) | 83 (5.9%) | 138 (9.9%) | <0.001 |
| Mortality, N (%) | 268 (4.8%) | 45 (3.2%) | 38 (2.7%) | 59 (4.2%) | 126 (9.0%) | <0.001 |
| Urgent heart TPL, N (%) | 70 (1.3%) | 16 (1.1%) | 15 (1.1%) | 26 (1.9%) | 13 (0.9%) | 0.119 |
| Hospital stay, median (IQR) | 9 (6–15) | 8 (6–13) | 8 (6–13) | 9 (6–15) | 11 (7–19) | <0.001 |
| ICU/CCU admission, N (%) | 2717 (48.7%) | 606 (43.4%) | 574 (41.1%) | 683 (49.0%) | 854 (61.2%) | <0.001 |

**Supplementary Table 2.** Univariate and multivariate logistic regression analyses for all-cause in-hospital and post discharge 3-years mortality in HF Patients subgroup analysis by de novo HF versus worsening of pre-existing HF.

|  | **De novo HF** | | | | **Worsening of chronic HF** | | | |
| --- | --- | --- | --- | --- | --- | --- | --- | --- |
|  | In-hospital mortality  (n = 2,911) | | Post discharge 3-year mortality  (n = 2,769) | | In-hospital mortality  (n = 2,669) | | Post discharge 3-year mortality  (n = 2,531) | |
|  | Unadjusted OR | Adjusted OR^a^ | Unadjusted OR | Adjusted OR^b^ | Unadjusted OR | Adjusted OR^a^ | Unadjusted OR | Adjusted OR^b^ |
| Quartile 1  (0.2-2.0) | 1.0  (Ref.) | 1.0  (Ref.) | 1.0  (Ref.) | 1.0  (Ref.) | 1.0  (Ref) | 1.0  (Ref) | 1.0  (Ref) | 1.0  (Ref) |
| Quartile 2  (2.1-3.2) | 0.47  (0.24–0.94) | 0.64  (0.27–1.49) | 1.25  (0.99–1.56) | 1.10  (0.87–1.39) | 1.32  (0.72–2.41) | 0.99  (0.52–1.89) | 1.34  (1.11–1.62) | 1.20  (0.99–1.47) |
| Quartile 3  (3.3-5.8) | 1.13  (0.65–1.97) | 1.34  (0.66–2.73) | 1.68  (1.36–2.09) | 1.18  (0.94–1.48) | 1.56  (0.88–2.77) | 0.97  (0.52–1.82) | 1.96  (1.65–2.34) | 1.57  (1.30–1.90) |
| Quartile 4  (5.9-192.4) | 2.80  (1.76–4.45) | 2.46  (1.28–4.74) | 2.18  (1.78–2.67) | 1.36  (1.09–1.70) | 3.22  (1.89–5.48) | 1.94  (1.07–3.52) | 2.45  (2.05–2.93) | 1.71  (1.40–2.08) |

Data are expressed as odds ratio (OR) and 95% confidence intervals (CI). Ref. = reference category.

^a^adjusted for age category (70> vs. 70≤), sex (male vs. female), body mass index category (25> vs. 23≤), etiology of heart failure (ischemic vs. non-ischemic), systolic blood pressure (100> vs. 100≤), history of hypertension, history of diabetes mellitus, history of cerebrovascular disease, history of chronic obstructive disease, prior admission history due to HF, presented tachyarrhythmia on admission, sodium level (135> vs. 135≤), creatinine level (2.0> vs. 2.0≤), left ventricular ejection fraction (HFrEF vs HFmrEF vs HFpEF).

^b^adjusted for age category (70> vs. 70≤), sex (male vs. female), body mass index category (23> vs. 23≤), etiology of heart failure (ischemic vs. non-ischemic), systolic blood pressure (100> vs. 100≤), history of hypertension, history of diabetes mellitus, history of cerebrovascular disease, history of chronic obstructive disease, prior admission history due to HF, presented tachyarrhythmia on admission, sodium level (135> vs. 135≤), creatinine level (2.0> vs. 2.0≤), left ventricular ejection fraction (HFrEF vs HFmrEF vs HFpEF), AA, BB, RASi

**Supplementary Table 3.** Univariate and multivariate logistic regression analyses for all-cause in-hospital and post discharge 3-years mortality in HF Patients subgroup analysis by LVEF categories.

|  | **HFrEF**  **(LVEF <40%)** | | | | **HFmrEF**  **(40% ≤ LVEF <50%)** | | | | **HFpEF**  **(LVEF ≥50%)** | | | |
| --- | --- | --- | --- | --- | --- | --- | --- | --- | --- | --- | --- | --- |
|  | In-hospital mortality  (n = 3,146) | | Post discharge 3-year mortality  (n = 2,928) | | In-hospital mortality  (n = 837) | | Post discharge 3-year mortality  (n = 796) | | In-hospital mortality  (n = 1,332) | | Post discharge 3-year mortality  (n = 1,272) | |
|  | Unadjusted  OR | Adjusted  OR^a^ | Unadjusted  OR | Adjusted  OR^b^ | Unadjusted  OR | Adjusted  OR^a^ | Unadjusted  OR | Adjusted  OR^b^ | Unadjusted  OR | Adjusted  OR^a^ | Unadjusted  OR | Adjusted  OR^b^ |
| Quartile 1  (0.2-2.0) | 1.0  (Ref.) | 1.0  (Ref.) | 1.0  (Ref.) | 1.0  (Ref.) | 1.0  (Ref.) | 1.0  (Ref.) | 1.0  (Ref.) | 1.0  (Ref.) | 1.0  (Ref.) | 1.0  (Ref.) | 1.0  (Ref.) | 1.0  (Ref.) |
| Quartile 2  (2.1-3.2) | 0.87  (0.50–1.52) | 0.79  (0.44–1.42) | 1.26  (1.04–1.53) | 1.06  (0.87–1.28) | 1.23  (0.27–5.55) | 1.07  (0.21–5.51) | 1.27  (0.87–1.87) | 1.17  (0.79–1.74) | 0.95  (0.29–3.14) | 1.00  (0.28–3.59) | 1.45  (1.08–1.96) | 1.30  (0.95–1.76) |
| Quartile 3  (3.3-5.8) | 1.40  (0.84–2.34) | 1.00  (0.57–1.75) | 1.84  (1.53–2.21) | 1.29  (1.07–1.56) | 1.53  (0.36–6.51) | 1.63  (0.36–7.33) | 1.84  (1.28–2.64) | 1.49  (1.02–2.17) | 1.93  (0.72–5.21) | 1.42  (0.47–4.36) | 2.00  (1.52–2.64) | 1.53  (1.15–2.04) |
| Quartile 4  (5.9-192.4) | 3.13  (1.99–4.91) | 2.00  (1.19–3.35) | 2.19  (1.83–2.63) | 1.36  (1.12–1.66) | 4.95  (1.44–17.07) | 4.96  (1.32–18.59) | 2.28  (1.62–3.23) | 1.60  (1.10–2.47) | 3.12  (1.21–8.01) | 1.83  (0.60–5.56) | 2.48  (1.89–3.26) | 1.81  (1.34–2.42) |

Data are expressed as odds ratio (OR) and 95% confidence intervals (CI). Ref. = reference category.

^a^adjusted for age category (70> vs. 70≤), sex (male vs. female), body mass index category (25> vs. 23≤), etiology of heart failure (ischemic vs. non-ischemic), systolic blood pressure (100> vs. 100≤), history of hypertension, history of diabetes mellitus, history of cerebrovascular disease, history of chronic obstructive disease, prior admission history due to HF, presented tachyarrhythmia on admission, sodium level (135> vs. 135≤), creatinine level (2.0> vs. 2.0≤), left ventricular ejection fraction (HFrEF vs HFmrEF vs HFpEF).

^b^adjusted for age category (70> vs. 70≤), sex (male vs. female), body mass index category (23> vs. 23≤), etiology of heart failure (ischemic vs. non-ischemic), systolic blood pressure (100> vs. 100≤), history of hypertension, history of diabetes mellitus, history of cerebrovascular disease, history of chronic obstructive disease, prior admission history due to HF, presented tachyarrhythmia on admission, sodium level (135> vs. 135≤), creatinine level (2.0> vs. 2.0≤), left ventricular ejection fraction (HFrEF vs HFmrEF vs HFpEF), AA, BB, RASi

**Supplementary Table 4.** In-hospital clinical outcomes of HF patients whose aggravating factor were not infection or ischemia stratified by quartiles of neutrophil to lymphocyte ratio at admission.

|  | **Total population** | **Neutrophil to lymphocyte ratio** | | | | |
| --- | --- | --- | --- | --- | --- | --- |
|  | (n = 3,127) | **Quartile 1**  (n = 782) | **Quartile 2**  (n = 783) | **Quartile 3**  (n = 781) | **Quartile 4**  (n = 781) | **P-value** |
| Total mortality, N (%)  (including urgent heart TPL) | 149 (4.8%) | 21 (2.7%) | 23 (2.9%) | 40 (5.1%) | 65 (8.3%) | <0.001 |
| Mortality, N (%) | 101 (3.2%) | 12 (1.5%) | 13 (1.7%) | 24 (3.1%) | 52 (6.7%) | <0.001 |
| Urgent heart TPL, N (%) | 52 (1.7%) | 10 (1.3%) | 12 (1.5%) | 16 (2.0%) | 14 (1.8%) | 0.664 |
| Hospital stay, median (IQR) | 9 (6–14) | 8 (5–12) | 8 (6–12) | 9 (6–14) | 11 (7–17) | <0.001 |
| ICU/CCU admission, N (%) | 1,218 (39.0%) | 274 (35.0%) | 266 (34.0%) | 292 (37.4%) | 386 (49.4%) | <0.001 |

**Supplementary Table 5.** In-hospital clinical outcomes of HF patients whose aggravating factor were infection or ischemia stratified by quartiles of neutrophil to lymphocyte ratio at admission.

|  | **Total population** | **Neutrophil to lymphocyte ratio** | | | | |
| --- | --- | --- | --- | --- | --- | --- |
|  | (n = 2,453) | **Quartile 1**  (n = 613) | **Quartile 2**  (n = 614) | **Quartile 3**  (n = 613) | **Quartile 4**  (n = 613) | **P-value** |
| Total mortality, N (%)  (including urgent heart TPL) | 182 (7.4%) | 38 (6.2%) | 24 (3.9%) | 51 (8.3%) | 69 (11.3%) | <0.001 |
| Mortality, N (%) | 167 (6.8%) | 34 (5.5%) | 23 (3.7%) | 44 (7.2%) | 66 (10.8%) | <0.001 |
| Urgent heart TPL, N (%) | 18 (0.7%) | 5 (0.8%) | 1 (0.2%) | 9 (1.5%) | 3 (0.5%) | 0.049 |
| Hospital stay, median (IQR) | 10 (6–16) | 8 (6–14) | 9 (6–14) | 10 (7–16) | 12 (8–21) | <0.001 |
| ICU/CCU admission, N (%) | 1,499 (61.1%) | 363 (59.2%) | 320 (52.1%) | 386 (63.0%) | 430 (70.1%) | <0.001 |

**Supplementary Table 6.** Univariate and multivariate logistic regression analyses for all-cause in-hospital and Cox regression analysis for post discharge 3-years mortality in HF Patients stratified by quartiles of neutrophil to lymphocyte ratio at admission.

|  | In-hospital mortality (n = 5,580) | | | | Post discharge 3 year mortality (n = 5,312) | | | |
| --- | --- | --- | --- | --- | --- | --- | --- | --- |
|  | Unadjusted OR | P-value | Adjusted OR^a^ | P-value | Unadjusted OR | P-value | Adjusted OR^b^ | P-value |
| Quartile 1 (0.2-2.0) | 1.0 (Ref.) |  | 1.0 (Ref.) |  | 1.0 (Ref.) |  | 1.0 (Ref.) |  |
| Quartile 2 (2.1-3.2) | 0.84 (0.54–1.30) | 0.436 | 0.86 (0.52–1.42) | 0.549 | 1.31 (1.13–1.51) | <0.001 | 1.11(0.96–1.29) | 0.172 |
| Quartile 3 (3.3-5.8) | 1.33 (0.89–1.97) | 0.163 | 1.17 (0.73–1.87) | 0.514 | 1.88 (1.64–2.16) | <0.001 | 1.35 (1.16–1.55) | <0.001 |
| Quartile 4 (5.9-192.4) | 2.98 (2.10–4.22) | <0.001 | 2.23 (1.44–3.44) | <0.001 | 2.27 (1.99–2.60) | <0.001 | 1.44 (1.24–1.67) | <0.001 |

Data are expressed as odds ratio (OR) and 95% confidence intervals (CI). Ref. = reference category.

^a^adjusted for age category (70> vs. 70≤), sex (male vs. female), body mass index category (25> vs. 23≤), etiology of heart failure (ischemic vs. non-ischemic), systolic blood pressure (100> vs. 100≤), history of hypertension, history of diabetes mellitus, history of cerebrovascular disease, history of chronic obstructive disease, prior admission history due to HF, presented tachyarrhythmia on admission, sodium level (135> vs. 135≤), creatinine level (2.0> vs. 2.0≤), left ventricular ejection fraction (HFrEF vs HFmrEF vs HFpEF), BNP ≥500 pg/mL or NT-proBNP ≥1,500 pg/mL

^b^adjusted for age category (70> vs. 70≤), sex (male vs. female), body mass index category (23> vs. 23≤), etiology of heart failure (ischemic vs. non-ischemic), systolic blood pressure (100> vs. 100≤), history of hypertension, history of diabetes mellitus, history of cerebrovascular disease, history of chronic obstructive disease, prior admission history due to HF, presented tachyarrhythmia on admission, sodium level (135> vs. 135≤), creatinine level (2.0> vs. 2.0≤), left ventricular ejection fraction (HFrEF vs HFmrEF vs HFpEF), BNP ≥500 pg/mL or NT-proBNP ≥1,500 pg/mL, AA, BB, RASi

**Supplementary Table 7.** Univariate and multivariate logistic regression analyses for all-cause in-hospital and post discharge 3-year mortality in HF Patients subgroup analysis by aggravating factor

1. **Patients whose aggravating factor were not infection or ischemia.**

|  | In-hospital mortality (n = 3,127) | | | | Post discharge 3 year mortality (n = 3,026) | | | |
| --- | --- | --- | --- | --- | --- | --- | --- | --- |
|  | Unadjusted OR | P-value | Adjusted OR^a^ | P-value | Unadjusted OR | P-value | Adjusted OR^b^ | P-value |
| Quartile 1 (0.2-1.9) | 1.0 (Ref.) |  | 1.0 (Ref.) |  | 1.0 (Ref.) |  | 1.0 (Ref.) |  |
| Quartile 2 (2.0-2.9) | 1.08 (0.49–2.39) | 0.843 | 0.85 (0.37–1.96) | 0.702 | 1.23 (1.00–1.50) | 0.049 | 0.98 (0.76–1.27) | 0.868 |
| Quartile 3 (3.0-4.9) | 2.03 (1.01–4.10) | 0.047 | 1.41 (0.67–2.96) | 0.365 | 1.92 (1.59–2.32) | <0.001 | 1.38 (1.07–1.77) | 0.014 |
| Quartile 4 (5.0-95.0) | 4.58 (2.42–8.64) | <0.001 | 2.39 (1.19–4.81) | 0.015 | 2.58 (2.15–3.10) | <0.001 | 1.81 (1.40–2.35) | <0.001 |

1. **Patients whose aggravating factor were infection or ischemia**

|  | In-hospital mortality (n = 2,453) | | | | Post discharge 3 year mortality (n = 2,286) | | | |
| --- | --- | --- | --- | --- | --- | --- | --- | --- |
|  | Unadjusted OR | P-value | Adjusted OR^a^ | P-value | Unadjusted OR | P-value | Adjusted OR^b^ | P-value |
| Quartile 1 (0.2-2.3) | 1.0 (Ref.) |  | 1.0 (Ref.) |  | 1.0 (Ref.) |  | 1.0 (Ref.) |  |
| Quartile 2 (2.4-3.8) | 0.66 (0.39–1.14) | 0.136 | 0.83 (0.43–1.62) | 0.590 | 1.22 (0.99–1.50) | 0.061 | 1.01 (0.77–1.34) | 0.971 |
| Quartile 3 (3.9-7.0) | 1.32 (0.83–2.09) | 0.243 | 1.65 (0.91–2.93) | 0.099 | 1.51 (1.24–1.84) | <0.001 | 1.18 (0.89–1.55) | 0.288 |
| Quartile 4 (7.1-192.4) | 2.06 (1.34–3.16) | 0.001 | 2.24 (1.26–4.00) | 0.006 | 1.93 (1.59–2.34) | <0.001 | 1.30 (1.05–1.73) | 0.027 |

Data are expressed as odds ratio (OR) and 95% confidence intervals (CI). Ref. = reference category.

^a^adjusted for age category (70> vs. 70≤), sex (male vs. female), body mass index category (25> vs. 23≤), etiology of heart failure (ischemic vs. non-ischemic), systolic blood pressure (100> vs. 100≤), history of hypertension, history of diabetes mellitus, history of cerebrovascular disease, history of chronic obstructive disease, prior admission history due to HF, presented tachyarrhythmia on admission, sodium level (135> vs. 135≤), creatinine level (2.0> vs. 2.0≤), left ventricular ejection fraction (HFrEF vs HFmrEF vs HFpEF), BNP ≥500 pg/mL or NT-proBNP ≥1,500 pg/mL

^b^adjusted for age category (70> vs. 70≤), sex (male vs. female), body mass index category (23> vs. 23≤), etiology of heart failure (ischemic vs. non-ischemic), systolic blood pressure (100> vs. 100≤), history of hypertension, history of diabetes mellitus, history of cerebrovascular disease, history of chronic obstructive disease, prior admission history due to HF, presented tachyarrhythmia on admission, sodium level (135> vs. 135≤), creatinine level (2.0> vs. 2.0≤), left ventricular ejection fraction (HFrEF vs HFmrEF vs HFpEF), BNP ≥500 pg/mL or NT-proBNP ≥1,500 pg/mL, AA, BB, RASi
