# Supplementary material for: Neutrophil-Lymphocyte Ratio in Patients with Acute Heart Failure Predicts In-Hospital and Long-Term Mortality
Source: J Clin Med. 2020 Feb 18;9(2):557. doi: 10.3390/jcm9020557 (PMC7073552; doi:10.3390/jcm9020557)
Supplement: Supplementary file 1 [file jcm-09-00557-s001.zip › NL_ratio_supple figure_JCM_revised_final.pptx]

## Slide 1
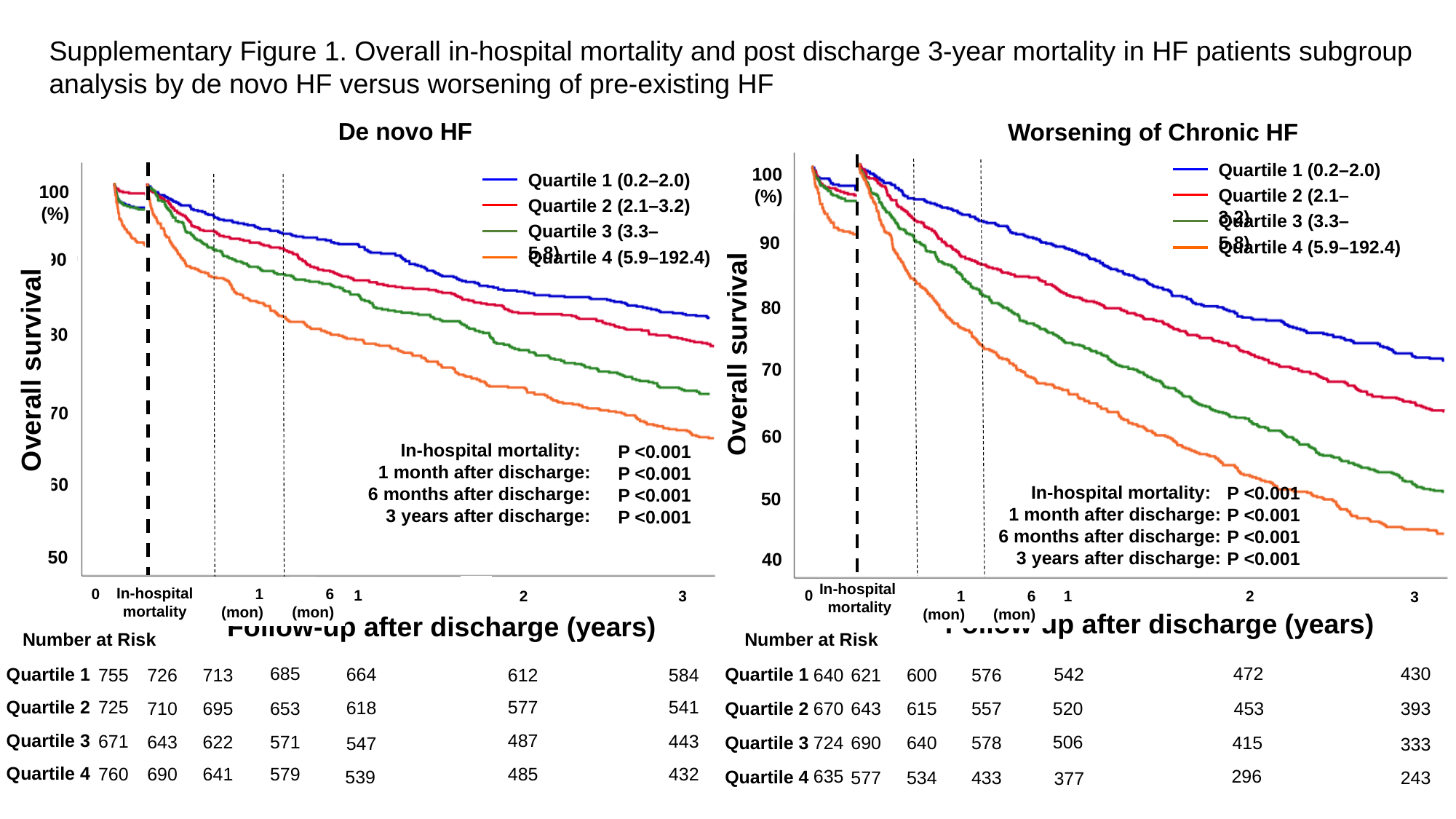

Supplementary Figure 1. Overall in-hospital mortality and post discharge 3-year mortality in HF patients subgroup analysis by de novo HF versus worsening of pre-existing HF
De novo HF
Worsening of Chronic HF
100
(%)
90
80
Overall survival
70
60
50
40
In-hospital
mortality
0
2
1
3
Quartile 1 (0.2–2.0)
100
(%)
90
80
Overall survival
70
60
50
In-hospital
mortality
0
1
2
3
Quartile 1 (0.2–2.0)
Quartile 2 (2.1–3.2)
Quartile 2 (2.1–3.2)
Quartile 3 (3.3–5.8)
Quartile 3 (3.3–5.8)
Quartile 4 (5.9–192.4)
Quartile 4 (5.9–192.4)
In-hospital mortality:
1 month after discharge:
6 months after discharge:
3 years after discharge:
P <0.001
P <0.001
P <0.001
P <0.001
In-hospital mortality:
1 month after discharge:
6 months after discharge:
3 years after discharge:
P <0.001
P <0.001
P <0.001
P <0.001
6 (mon)
1 (mon)
6 (mon)
1 (mon)
Follow-up after discharge (years)
Follow-up after discharge (years)
Number at Risk
Number at Risk
430
685
472
Quartile 1
Quartile 1
542
664
755
612
584
640
621
600
576
726
713
577
Quartile 2
725
541
618
453
Quartile 2
670
393
710
695
653
643
615
557
520
Quartile 3
487
671
443
643
622
571
506
415
Quartile 3
724
690
640
578
547
333
Quartile 4
485
760
432
690
641
579
296
635
Quartile 4
539
243
577
534
433
377

## Slide 2
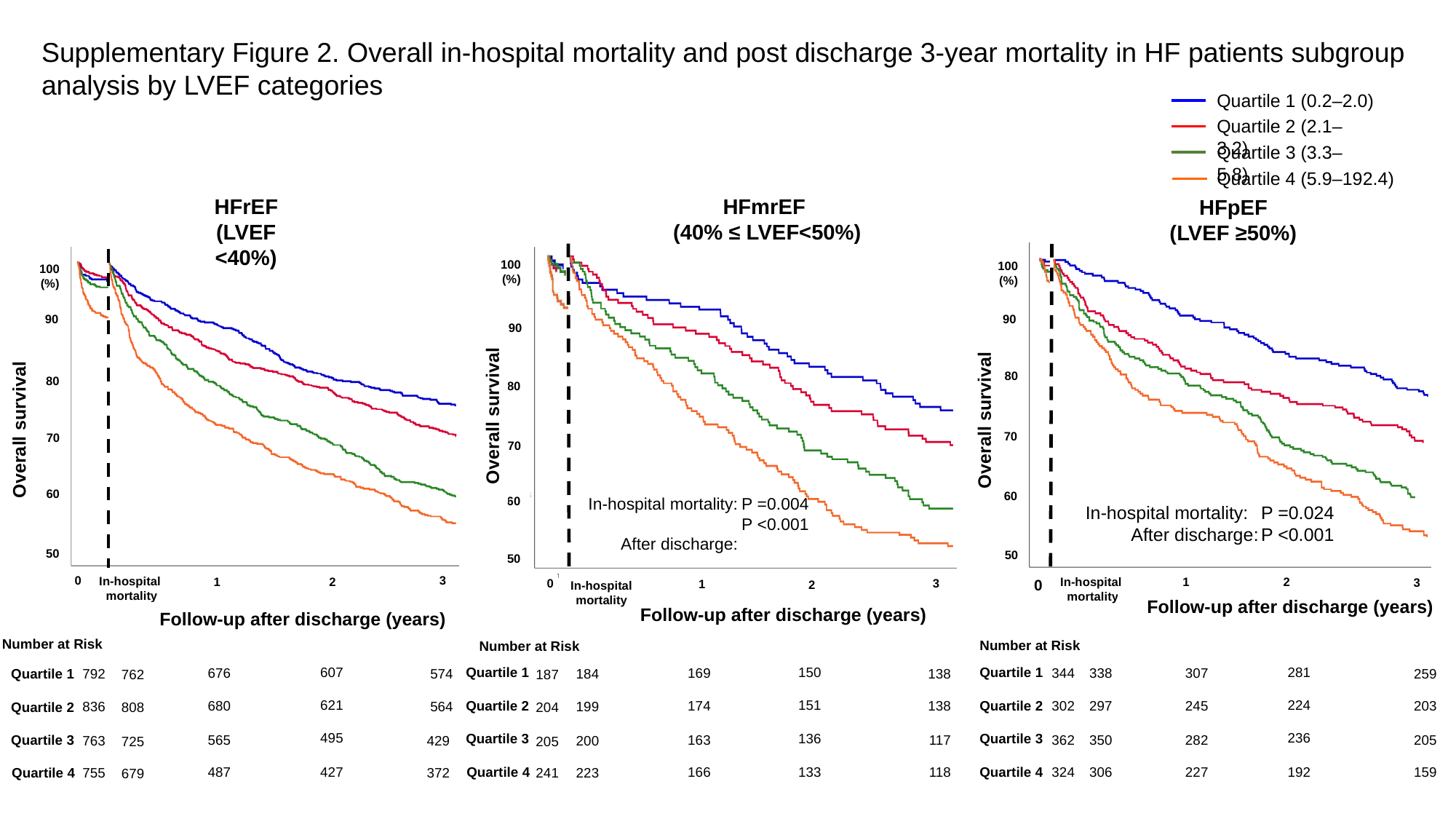

Supplementary Figure 2. Overall in-hospital mortality and post discharge 3-year mortality in HF patients subgroup analysis by LVEF categories
Quartile 1 (0.2–2.0)
Quartile 2 (2.1–3.2)
Quartile 3 (3.3–5.8)
Quartile 4 (5.9–192.4)
HFmrEF
(40% ≤ LVEF<50%)
HFrEF
(LVEF <40%)
HFpEF
(LVEF ≥50%)
100
(%)
90
80
Overall survival
70
60
50
3
0
1
2
In-hospital
mortality
Follow-up after discharge (years)
100
(%)
90
80
Overall survival
70
60
50
2
In-hospital
mortality
1
0
3
Follow-up after discharge (years)
P =0.024
P <0.001
In-hospital mortality:
After discharge:
100
(%)
90
80
Overall survival
70
60
50
0
3
In-hospital
mortality
1
2
In-hospital mortality:
After discharge:
P =0.004
P <0.001
Follow-up after discharge (years)
Number at Risk
Number at Risk
Number at Risk
607
281
Quartile 1
Quartile 1
150
676
169
344
338
307
259
138
Quartile 1
792
574
184
187
762
621
224
151
Quartile 2
Quartile 2
680
174
302
297
245
203
138
836
564
199
204
Quartile 2
808
495
236
136
Quartile 3
Quartile 3
Quartile 3
565
163
362
350
282
205
117
763
429
200
205
725
487
Quartile 4
166
Quartile 4
324
306
227
427
192
159
118
133
755
372
223
241
Quartile 4
679
